# Substitution of Glu378 in EF-Tu disrupts binding to KKL-55

**DOI:** 10.17912/micropub.biology.001254

**Published:** 2024-09-03

**Authors:** Michael Y. Vazquez Soba, Neeraja Marathe, Kenneth C. Keiler

**Affiliations:** 1 Pennsylvania State University, University Park, PA USA; 2 University of Texas at Austin, Austin, TX USA

## Abstract

*Trans* -translation is a target for the development of new antibiotics. The potential antibiotic lead compound KKL-55 binds to EF-Tu and inhibits *trans* -translation. Previous structural and biochemical studies showed that glutamate 378 in EF-Tu directly contacts bound KKL-55, but mutation of residue 378 to alanine had no effect on the equilibrium dissociation constant for binding of EF-Tu and KKL-55. Here, we found that a variant of EF-Tu with tryptophan at position 378 increases the *K _d_* for binding of EF-Tu and KKL-55 by >6-fold, indicating that a larger side chain at this position is disruptive. The E378W variant decreased the amount of translation *in vitro* and no *trans* -translation could be detected with this variant. These data provide further evidence that residue 378 of EF-Tu forms part of the KKL-55 binding pocket and are consistent with a lack of spontaneous mutants resistant to KKL-55.

**Figure 1. EF-Tu E378W decreases binding affinity and translational activity:**
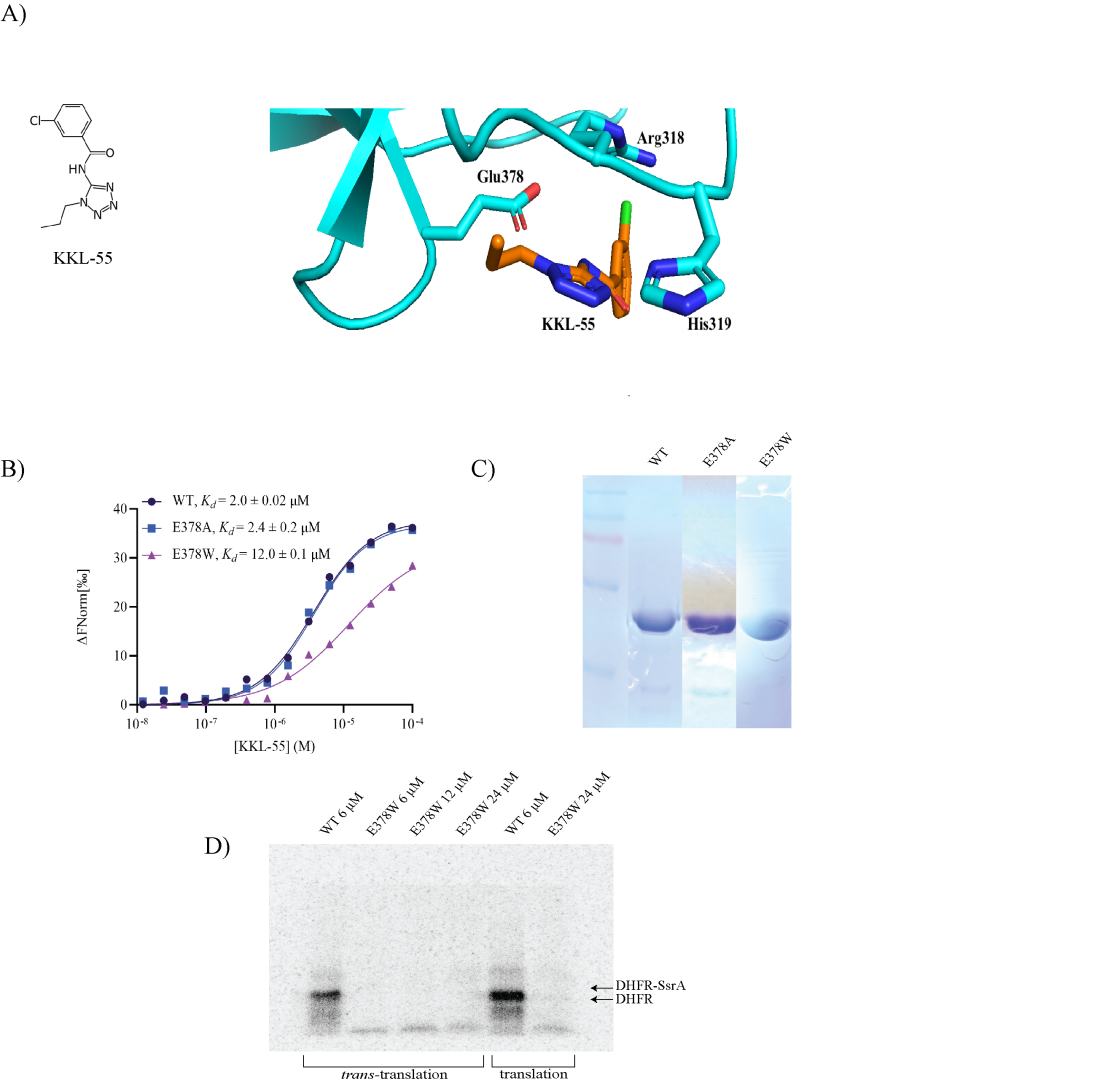
(A) KKL-55 structure and KKL-55 bound to domain 3 of EF-Tu (cyan) with binding pocket residues Glu378, Arg318, and His319 shown. (B) Microscale thermophoresis binding assays for wild-type and variant EF-Tu with KKL-55. The equilibrium dissociation constants were calculated by non-linear curve fitting. One representative binding curve for each protein is shown. Mean dissociation constant with standard deviation from at least 3 repeats is shown for each protein. (C) Coomassie Brilliant Blue stained SDS polyacrylamide gels showing purity of EF-Tu variants. Protein is overloaded to show contaminating bands. (D) *In vitro* *trans* -translation and translation reactions with wild-type EF-Tu and the E378W variant at different concentrations. The mobility of full-length DHFR from normal translation and DHFR-SsrA from *trans* -translation are indicated.

## Description

The emergence of multi-drug resistant pathogenic bacteria has stimulated the search and development for new therapeutic agents capable of mitigating this rising public health crisis. *Trans* -translation was identified as a potential candidate for the development of antibiotic therapies because many bacterial pathogens are reliant on it for survival and it is not present in metazoans (Ramadoss et al., 2013) . During translation, ribosomes can encounter errors in which decoding of mRNA lacking a stop codon at the 3’end leads to the formation of non-stop complexes (Keiler, & Feaga, 2014) . The *trans-* translation pathway uses transfer-messenger RNA (tmRNA), a molecule containing properties of transfer RNA and messenger RNA, in a complex with SmpB to rescue ribosomes stalled in non-stop complexes. tmRNA in a complex with EF-Tu and SmpB recognizes the A site of the stalled ribosome (Keiler, 2015) . Using the coding sequence within tmRNA, the growing polypeptide is tagged for degradation and the ribosome is disassembled, preventing the toxic accumulation of non-stop complexes (Keiler, 2015) .

KKL-55, a tetrazole-based small molecule, was identified as an inhibitor of *trans* -translation. The molecule binds EF-Tu and prevents the binding of tmRNA, but not tRNA (Marathe et al., 2023) . X-ray crystallography studies showed that KKL-55 interacts with EF-Tu at a pocket formed by residues Arg318, His319, and Glu378 ( Figure 1A ). Upon KKL-55 binding, the Glu378 residue reorients to face the binding pocket and interacts with KKL-55 (Marathe et al., 2023) . Mutation of Glu378 to alanine had little impact on the binding affinity between EF-Tu and KKL-55, raising the possibility that energy gained from interaction of Glu378 with KKL-55 was offset by energy lost in the conformational change (Marathe et al., 2023) . Nevertheless, if Glu378 forms part of the KKL-55 binding pocket, more disruptive mutations at this position would be expected to alter the binding affinity. In this study, we tested the effects of mutating Glu378 to tryptophan to assess whether a larger amino acid at this position would block interactions with KKL-55.

A site-directed mutation of residue 378 to tryptophan was constructed and the variant protein was purified. A micro-scale thermophoresis assay (MST) was used to investigate the affinity between the E378W variant and KKL-55 ( Figure 1B ). Consistent with prior studies (Marathe et al., 2023) , wild-type EF-Tu and KKL-55 bound with a *K _d_* = 2.0 µM, and EF-Tu E378A and KKL-55 bound with a *K _d_* = 2.4 µM. In contrast, the E378W mutant bound KKL-55 weakly, such that binding was not saturated at the highest concentration of KKL-55 that could be used without aggregation. To estimate the minimum boundary for the *K _d_* , the binding curve was fit assuming saturation at the highest concentration of KKL-55, and under these conditions *K _d_* = 12.0 ± 0.1 µM. These data indicate that binding affinity was >6-fold weaker for the E378W variant than for wild-type EF-Tu. Such changes in the binding affinity are consistent with Glu378 forming part of the KKL-55 binding pocket.

Based on the binding affinity *in vitro* , bacteria might become resistant to KKL-55 through mutations to Glu378. However, no resistant mutants have been isolated (Marathe et al., 2023) . To determine whether EF-Tu E378W could replace wild-type EF-Tu in protein synthesis, translation and *trans-* translation activities were assayed *in vitro* ( Figure 1D ). The amount of translation was dramatically lower when EF-Tu E378W was used as compared to wild-type EF-Tu. Only 5% of full-length DHFR was produced in reactions with the E378W variant even though it was added at up to 4-fold higher concentration. No *trans* -translation could be observed using the E378W variant ( Figure 1D ). The decreased translational activity suggests that the E378W mutation would not result in resistance to KKL-55 because the mutant cells are unlikely to be viable. Prior studies of an EF-Tu E378A mutant showed that Glu378 has a substantial role in optimizing the affinity of tRNAs and EF-Tu to maximize protein synthesis (Schrader et al., 2011) . The interactions between Glu378 and tRNAs could explain why we observed a decrease in translational activity in the E378W mutant. Analysis of other prokaryotic EF-Tu protein sequences homologous to *E. coli* K12 EF-Tu showed a strict conservation of the Glu378 residue in the KKL-55 binding pocket (Marathe et al., 2023) . Retaining the interaction between Glu378 and KKL-55 might be beneficial during optimization of the antibiotic activity of KKL-55 if this interaction limits the potential for evolution of resistant mutants.

## Methods

**Site-directed mutagenesis of EF-Tu.** The E378W mutation was constructed using a 3-piece assembly strategy as previously described for the E378A mutation (Marathe et al., 2023) . Briefly, two PCR products were made from a pCA24N-His6-tufA template using primers ACCATCACCATCACCCATACGTCTAAAGAAAAATTTGAACGTACAAAACCGCACGTTAAC and GGTACGGCCGCCCCAACGGATTGCGAA for one fragment and GCTGCAGGTCGACCCTTAGCTTAGCCCAGAACTTTAGCAACAACGCCC and TTCGCAATCCGTTGGGGCGGCCGTACC for the other. These two fragments were assembled into pCA24N-His6-tufA which had been digested with BamHI and NotI, using HiFi (New England Biolabs, Ipswich, MA, USA).

**EF-Tu Expression and Purification.** *E. coli* DH5α with pCA24N-His6-tufA or pCA24N-His6-tufA(E378W) were grown in 1 L terrific broth (24 g/L yeast extract, 20 g/L tryptone, 4 mL/L glycerol, 0.017 M KH _2_ PO _4_ , 0.072 M K _2_ HPO _4_ ) at 37 ºC until the OD _600_ reached 0.6. 1 mM isopropyl-thio-β-d-galactoside (IPTG) was added and cells were grown for an additional 3 h to over-express the 6-His-tagged EF-Tu. Cells were harvested by centrifugation at 6953 *g* for 10 min, resuspended in lysis buffer (50 mM Tris-HCl pH 7.6, 60 mM NH _4_ CI, 7 mM MgCl _2_ , 7 mM β-Me, 15% [by vol] glycerol, 10 µM guanosine diphosphate, 10 mM imidazole, 1 mM phenylmethylsulfonyl fluoride) and lysed by sonication. The lysate was cleared by centrifugation at 28,000 *g* for 15 min and incubated with 750 µL HisPur Ni-NTA agarose resin (ThermoFisher, Waltham, MA USA) for 1 h. Resin was washed twice with 50 ml buffer A (50 mM Tris-HCl pH 8.0, 60 mM NH _4_ CI, 7 mM MgCl _2_ , 7 mM β-Me, 15% [by vol] glycerol, 10 µM GDP, 10 mM imidazole, 300 mM KCl), followed by two washes with buffer B (50 mM Tris-HCl pH 7.0, 60 mM NH _4_ CI, 7 mM MgCl _2_ , 7 mM β-Me, 15% [by vol] glycerol, 10 µM GDP, 10 mM imidazole, 300 mM KCl), loaded onto a column, and eluted with elution buffer (50 mM Tris-HCl pH 7.6, 60 mM NH _4_ CI, 7 mM MgCl _2_ , 7 mM β-Me, 15% [by vol] glycerol, 10 µM GDP, 500 mM imidazole). Fractions containing EF-Tu were pooled, dialyzed in buffer C (50 mM Tris-HCl pH 7.6, 60 mM NH _4_ CI, 7 mM MgCl _2_ , 7 mM β-Me, 15% [by vol] glycerol, 10 µM GDP), and stored in 50% glycerol with 20 µM GDP.

***In vitro* translation and *trans* -translation assays.** Assays were performed as described in previous studies (Marathe et al., 2023) . Briefly, the in-house *in vitro* protein synthesis kit was used to monitor protein synthesis through ^35^ S-methionine incorporation from a DHFR template. *In vitro trans* -translation was assessed in a reaction that additionally contained tmRNA-SmpB and a DHFR template missing two bases from the stop codon, as previously described (Marathe et al., 2023) . Removal of bases allows for the formation of a non-stop complex in which tmRNA-SmpB can then introduce an 11 amino acid tag on the DHFR protein. 75 µM, 150 µM, and 300 µM concentrations of EF-Tu•GDP were used for the reaction. Separation of the reaction cocktails via SDS-PAGE then allows quantification of tagged DHFR and untagged DHFR.

**Microscale thermophoresis-binding assays.** Assays were performed as previously described (Marathe et al., 2023) . Briefly, 6His-tagged EF-Tu•GDP (300 nM) was incubated with 75 nM Red Tris NTA dye Generation 2 (NanoTemper Technologies, Watertown, MA, USA) in binding buffer (287 mM NaCl, 2.7 mM KCl, 10 mM Na _2_ HPO _4_ , 1.8 mM KH _2_ PO _4_ , 0.01% Tween 20) for 30 min. The labeled EF-Tu•GDP was combined with KKL-55 in a 1:1 ratio and incubated at room temperature for 2 h. MST was measured in a Monolith NT.115 (NanoTemper Technologies, Watertown, MA, USA). Plots of change in fluorescence vs the concentration of KKL-55 were fit to the hyperbolic function *y* = *c* /(1 + *K*_d_ / *x* ) to obtain the apparent binding constant, as previously described, except that for the E378W variant the minimum *K _d_* was estimated by fixing the upper baseline at the value for 100 µM KKL-55 (Marathe et al., 2023) .
